# Effect of ischemic and pharmacological preconditioning of lower limb muscle tissue on tissue oxygenation measured by near-infrared spectroscopy – a pilot study

**DOI:** 10.1186/1471-2253-14-54

**Published:** 2014-07-15

**Authors:** Axel Fudickar, Sarah Kunath, Dana Voß, Markus Siggelkow, Erol Cavus, Markus Steinfath, Berthold Bein

**Affiliations:** 1 Department of Anesthesiology and Intensive Care Medicine, University Hospital Schleswig-Holstein, Campus Kiel, Schwanenweg 21, Kiel D-24105, Germany; 2 Department of Cardiovascular Surgery, University Hospital Schleswig-Holstein, Campus Kiel, Kiel, Germany

**Keywords:** Ischemic preconditioning, Sevoflurane, Surgical revascularization, Arterial occlusive disease

## Abstract

**Background:**

Ischemic or volatile anesthetic preconditioning is defined as tissue protection from impending ischemic cell damage by repetitive short periods of tissue exposure to ischemia or volatile anesthetics. Objective of this study was to elucidate, if ischemic preconditioning and pharmacological preconditioning with sevoflurane have effects on muscle tissue oxygen saturation in patients undergoing surgical revascularization of the lower limb.

**Methods:**

In this prospective randomized pilot study ischemic and pharmacological (sevoflurane) preconditioning was performed in 40 patients with lower limb arterial occlusive disease undergoing surgical revascularization. Sevoflurane preconditioning was performed in one group (N = 20) by repetitive application of sevoflurane for six minutes interspersed by six minutes of washout. Thereafter, ischemic preconditioning was performed in all patients (N = 40) by repetitive clamping of the femoral artery for six minutes interspersed by six minutes of reperfusion. The effect of both procedures on leg muscle tissue oxygen saturation (rSO_2_) was measured by near-infrared spectroscopy during both procedures and during surgery and reperfusion (INVOS® 5100C Oxymeter with Small Adult SomaSensor® SAFB-SM, Somanetics, Troy, Michigan, USA).

**Results:**

Repetitive clamping and reperfusion of the femoral artery resulted in significant cyclic decrease and increase of muscle rSO_2_ (p < 0.0001). Pharmacological preconditioning with sevoflurane resulted in a faster and higher increase of rSO_2_ during postoperative reperfusion (Maximal 111% baseline ± 20 versus 103% baseline ± 14, p = 0.008) consistent with an additional effect of pharmacological preconditioning on leg perfusion.

**Conclusions:**

Ischemic preconditioning of lower limb muscle tissue and pharmacological preconditioning with sevoflurane have an effect on tissue oxygenation in patients with lower limb occlusive arterial disease.

**Trial registration:**

The trial has been registrated at http://www.ClinicalTrial.gov, Trial Number: NCT02038062 at 14 January 2014.

## Background

Surgical revascularization of lower limb peripheral occlusive arterial disease is performed, if non-surgical procedures are not sufficient to treat lower limb ischemia [1]. Surgery for peripheral arterial disease of the leg is usually performed after clamping the femoral artery, thereby putting the lower limb at risk of ischemia and reperfusion damage. Reperfusion injury may also affect heart, lung and other organs remote from the ischemic tissue by release of ischemic metabolites during reperfusion.

Ischemic preconditioning (IPC) is defined as protection from ischemic cell damage by preceding repetitive short periods of ischemia followed by reperfusion [2]. IPC may also be achieved by repetitive ischemia of an organ or limb remote from the protected tissue (Remote ischemic preconditioning, RIPC) [3].

It has been shown that chronic ischemia of leg tissue can decrease myocardial infarct size and improve left ventricular function by increasing coronary collateral vessel density and blood flow probably by RIPC [4]. Hence, IPC by repetitive clamping of the femoral artery before surgery may protect lower limb tissue from ischemic and reperfusion injury during and after clamping of the femoral artery for vascular surgery and may also protect remote organs as the heart by RIPC. However, patients with limb ischemia due to occlusive arterial disease develop collateral vessels to the femoral artery. Due to the great variability of these vessels bypassing the femoral artery, it remains unclear if effective ischemia and reperfusion cycles can be achieved by clamping the femoral artery [5].

Preconditioning by sevoflurane is a pharmacological alternative to IPC. Patients who received sevoflurane for anesthesia during cardiac surgery had decreased postoperative mortality, less myocardial infarction and shorter length of hospital stay [6,7].

Near infrared spectroscopy (NIRS) enables non-invasive continuous measurement of tissue oxygation. NIRS is predominantly used for monitoring brain tissue oxygen saturation during cardiac surgery and a decrease by more than 20% from baseline is regarded as critical [8,9]. NIRS has also been used to measure rSO_2_ in the lower limb and to show the correlation of age and tissue perfusion in the anterior tibial muscle with sufficient sensitivity to perfusion changes [10]. NIRS showed also good reproducibility during ischemia and reperfusion of the forearm [11] and has been claimed to be useful for the non-invasive measurement of limb perfusion [12,13]. NIRS has been used to measure changes in regional tissue oxygen saturation (rSO_2_) in the calf muscle of patients with intermittent claudication during treadmill tests [14]. In another study the effect of medication on tissue perfusion in patients with occlusive arterial disease could be demonstrated by NIRS [15].

Hence, NIRS is regarded as an appropriate method to measure the effect of IPC and sevoflurane preconditioning on lower limb tissue oxygenation.

First purpose of this trial was to investigate, if repetitive clamping of the femoral artery results in repetitive decrease of leg tissue rSO_2_. Repetitive decrease of leg tissue rSO_2_ was regarded as a prerequisite of effective ischemic preconditioning of the lower limb in patients with occlusive arterial disease. Hence, we used NIRS to investigate the primary hypothesis that repetitive clamping of the femoral artery results in effective ischemic preconditioning of the lower limb in patients with occlusive arterial disease. Effective ischemic preconditioning was defined as two cycles of a statistically and clinically significant decrease and increase of tissue oxygen saturation measured by NIRS.

Second purpose of the trial was to examine, if sevoflurane preconditioning of the lower limb would probably be effective in patients with occlusive arterial disease. Effective sevoflurane preconditioning was defined as a significant effect of sevoflurane preconditioning on rSO_2_ measured by NIRS during IPC and on rSO_2_ measured by NIRS during postoperative reperfusion.

Repetitive decrease of leg rSO_2_ during repetitive clamping of the femoral artery and measurable effects of sevoflurane preconditioning on leg rSO_2_ would justify the design of a large randomized study to investigate the effects of ischemic and sevoflurane preconditioning on clinical outcome.

## Methods

### Subject enrolment

Ethics committee approval was obtained by the Ethics Committee of the University Hospital Schleswig-Holstein, Campus Kiel, Schwanenweg 20, 24105 Kiel, Germany. Written informed consent was obtained from all patients. Between May 2010 and January 2012 40 adult patients undergoing elective surgical treatment of peripheral occlusive arterial disease with clamping of the femoral artery under general anesthesia at the University Hospital Schleswig-Holstein, Campus Kiel were enrolled in this prospective randomized controlled interventional pilot study. Randomization was performed by putting 40 cards (20 cards each were labelled with the respective group assignment) in envelopes, mixing the envelopes randomly and numbering them consecutively.

Patients with skin disease rendering NIRS impossible and patients with amputation of the leg opposite to the side of surgery were excluded from the study.

### Induction of anesthesia and surgery

All patients received midazolam (7.5 mg, Hoffmann-La Roche, Basel, Switzerland) orally 30 minutes preoperatively for anxiolysis and sedation. Upon arrival in the anesthesia induction room, ECG, pulse oximetry and non-invasive blood pressure were monitored continuously.

Measurement of rSO_2_ was performed continuously from arrival at the anesthesia induction room until 20 minutes after postoperative declamping of the femoral artery using an INVOS® 5100C Oxymeter (Somanetics, Troy, Michigan, USA). The optodes (Small Adult SomaSensor® SAFB-SM, Somanetics, Troy, Michigan, USA) were fixed above the anterior tibial muscle at both lower legs 20 cm above the ankle after cleaning the skin with 70% ethanol. rSO_2_ of both legs was measured to exclude an oxygen saturation decrease or increase due to a systemic effect on leg tissue oxygen saturation during clamping.

The anesthetist performing anesthesia was blinded to the measurement of rSO_2_. Peripheral venous blood samples were obtained from the cubital vein for the measurement of hemoglobine, hematocrit, lactate, glucose, potassium, pO_2_, pCO_2_ and base excess immediately before induction of anesthesia.

Anesthesia was induced with intravenous propofol (2 mg.kg^-1^, Diprivan®, AstraZeneca, London, GB) and a continuous infusion of remifentanil (0.3 μg.kg^-1^.min^-1^, Ultiva®, GlaxoSmithKline, Brentford, GB). Muscle relaxation was achieved with rocuronium (0.6 mg.kg^-1^, Esmeron®, Essex Pharma, Oss, The Netherlands). After endotracheal intubation, ventilation was adjusted to maintain normocapnia (FiO_2_ = 0.3). Anesthesia was maintained with propofol (4–6 mg.kg^-1^.h^-1^) and remifentanil (0.2 - 0.3 μg.kg^-1^.min^-1^). Body temperature was kept normal by use of a heating blanket (36.5° - 37.5°C). All patients received cefuroxime (1.5 g) for antibiotic prophylaxis.

As soon as steady state anesthesia was achieved, sevoflurane preconditioning was performed in the sevoflurane group (N = 20) by two periods of sevoflurane application (each lasting 6 minutes) interspersed by 6 minutes washout on the basis of a previously published protocol for interrupted administration of sevoflurane for myocardial preconditioning [16].

Sevoflurane was administered with high fresh gas flow (10 l.min^-1^, FiO_2_ = 0.3). After an endtidal concentration of 1.0 MAC was reached, fresh gas flow was reduced to 4 l.min^-1^. rSO_2_ was noted immediately before application of sevoflurane and 1, 3 and 5 minutes thereafter. One minute after the last noted rSO_2_, the propofol infusion was resumed and sevoflurane was washed out by discontinuation of sevoflurane and increasing the fresh gas flow to 10 l.min^-1^ to achieve a MAC value below 0.2. rSO_2_ was noted immediately before washout and 1, 3 and 5 minutes thereafter. One minute after the last noted rSO_2_ during washout, application of sevoflurane and washout were repeated once. Akrinor® (0.5 ml cafedrine hydrochloride (100 mg.ml^-1^)/theodrenaline hydrochloride (10 mg.ml^-1^)) was given intravenously, if the systolic blood pressure decreased below 80% of baseline. In the other patients (IPC only, n = 20), rSO_2_ was measured during a respective time-matched period without sevoflurane preconditioning. These patients were anesthetized for the same period of time as the sevoflurane group.

After final washout of sevoflurane, IPC was performed in both groups by clamping the femoral artery at the side of surgery for 6 minutes. Clamping was performed using the same clamp (Pilling® Cooley Anastomosis Clamp, Teleflex® Medical, New York, USA) and the same clamping technique, as for definite clamping for surgery. rSO_2_ was noted immediately before clamping and 1, 3 and 5 minutes thereafter. One minute after the last noted rSO_2_ during clamping, the reperfusion period was started by declamping. rSO_2_ was noted immediately before declamping and 1, 3 and 5 minutes thereafter. One minute after the last rSO_2_ during reperfusion, clamping and reperfusion were repeated once.

Before the first clamping, intravenous heparin (100 units.kg^-1^, Heparin-Natrium-5000-Ratiopharm®, Ratiopharm, Ulm, Germany) was given to all patients. After the second reperfusion period, the femoral artery was definitely clamped for surgery. rSO_2_ was noted immediately after clamping for surgery, every 15 minutes during surgery, at declamping and subsequently at 3, 5, 10 and 20 minutes. After declamping, the heparin effect was partially reversed by intravenous protamine (60 – 80% of the initial heparin dose, MEDA Pharma, Bad Homburg, Germany). Immediately after extubation, venous blood samples were obtained from the cubital vein for the measurement of postoperative hemoglobine, hematocrit, lactate, glucose, potassium, pO_2_, pCO_2_ and base excess.

### Statistical analysis

Data analysis was performed with commercially available software (Graph Pad Prism® for Mac, version 5.03, GraphPad Software, San Francisco, USA).

The data were tested for normality using the Kolmogorov-Smirnov test. The influence of time and an additional factor (the side (left or right) or the medical preconditioning (with or without sevoflurane)) was analyzed by an ANOVA with repeated measurements with time as within subjects factor and side or medical preconditioning as between subjects factor.

The results were corrected for multiple testing. Parametric data are given as mean ± standard deviation. Differences between two groups were analyzed by the *t*-test for parametric data. P < 0.05 was considered statistically significant. Incidences of diagnoses, medications and localizations of stenoses were compared with Fisher’s exact test. Differences between laboratory values obtained before and after surgery and correlations of age, hemoglobine, ankle brachial index, sex, hyperlipidemia, diabetes mellitus and statins with rSO_2_ were investigated exploratively without correction for multiple testing.

If a medium effect size of f = 0.25, a medium correlation of 0.5 among repeated measures and a nonsphericity correction of 1 is assumed, the following power estimates apply for a significance level of 0.05 (calculated by G*Power 3.1.6): For all four time periods (sevoflurane preconditioning, ischemic preconditioning, clamping for surgery and postoperative reperfusion) the power for detecting a time effect was >99% and the power for detecting a side effect was about 85%. The power for detecting a medication effect varied between 38% (clamping for surgery), 54% (postoperative reperfusion) and 56% (sevoflurane preconditioning and ischemic preconditioning). The power for detecting an interaction between time and side or medication effect was for all time periods >99%.

## Results

### Demographic data and characteristics of patients

Demographic data and diagnoses are presented in Table 1. Most patients (n = 28) had peripheral occlusive arterial disease with Fontaine grade IIb. Medications and localizations of stenoses leading to surgery are shown in Table 2. Most frequent localizations were the superficial femoral artery (n = 27), the common femoral artery (n = 21) and the external iliac artery (n = 13).

**Table 1:** Demographic data, diagnoses and characteristics of patients

**Demographic data and diagnoses**	**IPC + sevoflurane (N = 20)**	**IPC (N = 20)**
Age (years)	68 ± 12	68 ± 11
Height (cm)	170 ± 11	170 ± 8
Weight (kg)	77 ± 17	75 ± 9
Male/female	14/6	13/7
Body-Mass-Index (BMI; kg.m^-2^)	27 ± 5	26 ± 3
Ankle-Brachial-Index (ABI)*	0.58 ± 0.18	0.56 ± 0.05
Side of surgery (right/left)	13 (65%)/7 (35%)	6 (30%)/14 (70%)
Fontaine grade (I/IIa/IIb/III/IV/V)	0/3/14/1/2 (0%/15%/70%/5%/10%)	0/5/14/0/1 (0%/25%/70%/0%/10%)
Obesity	5 (25%)	1 (5%)
Alcohol abuse	1 (5%)	1 (5%)
History of stroke	4 (20%)	1 (5%)
Diabetes mellitus	6 (30%)	5 (25%)
Hyperlipidaemia	14 (70%)	12 (60%)
Hypertonia	18 (90%)	17 (85%)
Hyperuricaemia	1 (5%)	5 (25%)
Coronary heart disease	9 (45%)	6 (30%)
Renal insufficiency	4 (20%)	1 (5%)
Smoking	9 (45%)	10 (50%)

*The “Ankle Brachial Index (ABI)” is defined as the systolic blood pressure measured at the ankle divided by the systolic blood pressure measured at the arm [17].

Demographic data, diagnoses and characteristics of patients. Data are given as mean ± standard deviation or absolute numbers (Percent). Differences between groups are not significant (“IPC + sevoflurane” = patients with sevoflurane preconditioning before ischemic preconditioning (IPC), “IPC” = patients without sevoflurane preconditioning before ischemic preconditioning).

**Table 2:** Medications of patients and localizations of stenoses

**Medication and localization of stenoses**	**IPC + sevoflurane (N = 20)**	**IPC (N = 20)**
Angiotensin converting enzyme inhibitor	9 (45%)	14 (70%)
Acetylsalicylic acid	16 (80%)	14 (70%)
Angiotensine 1 antagonist	4 (20%)	1 (5%)
β-adrenergic inhibitor	13 (65%)	13 (65%)
Calcium channel blocking agents	8 (40%)	5 (25%)
Diuretics	9 (45%)	4 (20%)
Digitalis	0 (0%)	3 (15%)
Insulin	5 (15%)	1 (5%)
Oral antidiabetics	2 (10%)	4 (20%)
Statins	8 (40%)	10 (50%)
Platelet aggregation inhibitor	2 (10%)	2 (10%)
Superficial femoral artery	14 (70%)	13 (65%)
Common femoral artery	12 (60%)	9 (45%)
External iliac artery	7 (35%)	6 (30%)
Common iliac artery	4 (20%)	5 (25%)
Profunda femoral artery	2 (10%)	2 (10%)
Internal iliac artery	1 (5%)	0 (0%)

Medications of patients and localization of stenoses in 40 patients undergoing surgical revascularization of the lower limb due to peripheral occlusive arterial disease. Data are given as absolute numbers (percent). Differences between groups are not significant. Total number of localisations is greater than 40, because there was more than one stenosis in some patients. (“IPC + sevoflurane” = patients with sevoflurane preconditioning before ischemic preconditioning (IPC), “IPC” = patients without sevoflurane preconditioning before ischemic preconditioning).

### Perioperative tissue oxygen saturation (rSO_2_)

#### *Baseline rSO_2_*

Mean baseline rSO_2_ of all patients was 64% ± 11 on the side of surgery and 65% ± 11 on the opposite side (Difference not significant). There was no significant difference of mean baseline rSO_2_ between patients with sevoflurane preconditioning and the control group (66% ± 12 vs. 62% ± 10). After induction of anesthesia, mean rSO_2_ of all patients was 66% ± 12 at the side of surgery and 66% ± 11 at the opposite side.

#### *Sevoflurane preconditioning*

**Figure 1:**
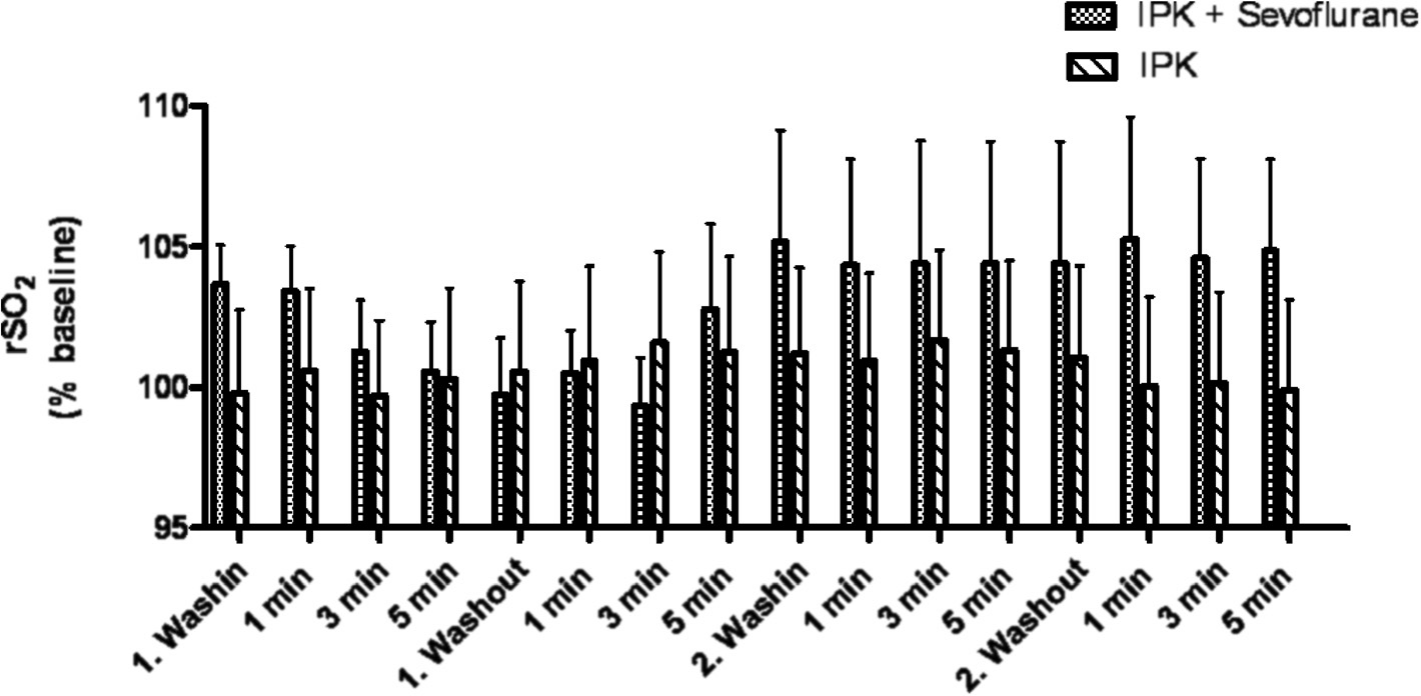
**rSO**_**2 **_**in the anterior tibial muscle during sevoflurane preconditioning and in a time-matched interval.** rSO_2_ in the anterior tibial muscle of patients during sevoflurane preconditioning (IPC + Sevoflurane, n = 20) and rSO_2_ of patients without sevoflurane preconditioning (IPC, n = 20) during a corresponding time-matched interval between induction of anesthesia and ischemic preconditioning. There was no significant difference between the groups. rSO_2_ has been normalized to baseline prior to induction of anesthesia to facilitate group comparison.

Figure 1 shows rSO_2_ in patients during sevoflurane preconditioning (n = 20) and in the group with IPK only during the corresponding time interval of surgery (n = 20). There was no significant effect of the factors “time” (p = 0.73), “sevoflurane” (p = 0.54) and the combination of the factors (p = 0.67) on rSO_2_ during sevoflurane preconditioning (Table 3). There was no significant difference between the time course of rSO_2_, on the side of surgery and the opposite side (control, no IPK) during sevoflurane preconditioning (p = 0.37, Table 4). Blood pressure and heart rate remained unchanged during sevoflurane preconditioning.

**Table 3:** Effects of time and sevoflurane preconditioning on lower limb tissue oxygenation during sevoflurane preconditioning, ischemic preconditioning, clamping for surgery and postoperative reperfusion

	**Sevoflurane preconditioning**	**Ischemic preconditioning**	**Clamping for surgery**	**Postoperative reperfusion**
Time	0.73	< 0.0001*	< 0.0001*	< 0.0001*
Sevoflurane	0.54	0.95	0.34	0.7
Interaction of “Time”with “Sevoflurane”	0.67	< 0.0001*	0.68	0.008*

Effects of the factors “time” and “sevoflurane preconditioning (sevoflurane)” and the interaction of “Time” (time from the beginning of sevoflurane preconditioning, ischemic preconditioning, clamping for surgery or postoperative reperfusion) with “sevoflurane” (sevoflurane preconditioning before ischemic preconditioning) on tissue oxygen saturation during sevoflurane preconditioning, ischemic preconditioning, clamping for surgery and postoperative reperfusion (ANOVA with repeated measurements with side as inter subject factor). Tissue oxygen saturation was measured with near-infrared spectroscopy in the anterior tibial muscle. Significant effects are marked with asterisks (*).

**Table 4:** Effect of time and side of measurement (side of surgery versus opposite side) on lower limb tissue oxygen saturation during sevoflurane preconditioning, ischemic preconditioning, clamping for surgery and postoperative reperfusion

	**Sevoflurane preconditioning**	**Ischemic preconditioning**	**Clamping for surgery**	**Postoperative reperfusion**
Time	0.61	< 0.0001*	< 0.0001*	< 0.0001*
Side	0.37	0.0007*	0.0001*	0.0027*
Interaction of “Side”with “Time”	0.74	< 0.0001*	< 0.0001*	< 0.0001*

Effects of the factors “time” (time from the beginning of sevoflurane preconditioning, ischemic preconditioning, clamping for surgery or psotoperative reperfusion) and “side” (side of surgery versus opposite side) on lower limb tissue oxygen saturation during sevoflurane preconditioning, ischemic preconditioning, clamping for surgery and postoperative reperfusion (ANOVA with repeated measurements with side as inter subject factor). Tissue oxygenation was measured with near-infrared spectroscopy in the anterior tibial muscle. Significant effects are marked with asterisks (*).

#### *Ischemic preconditioning*

**Figure 2:**
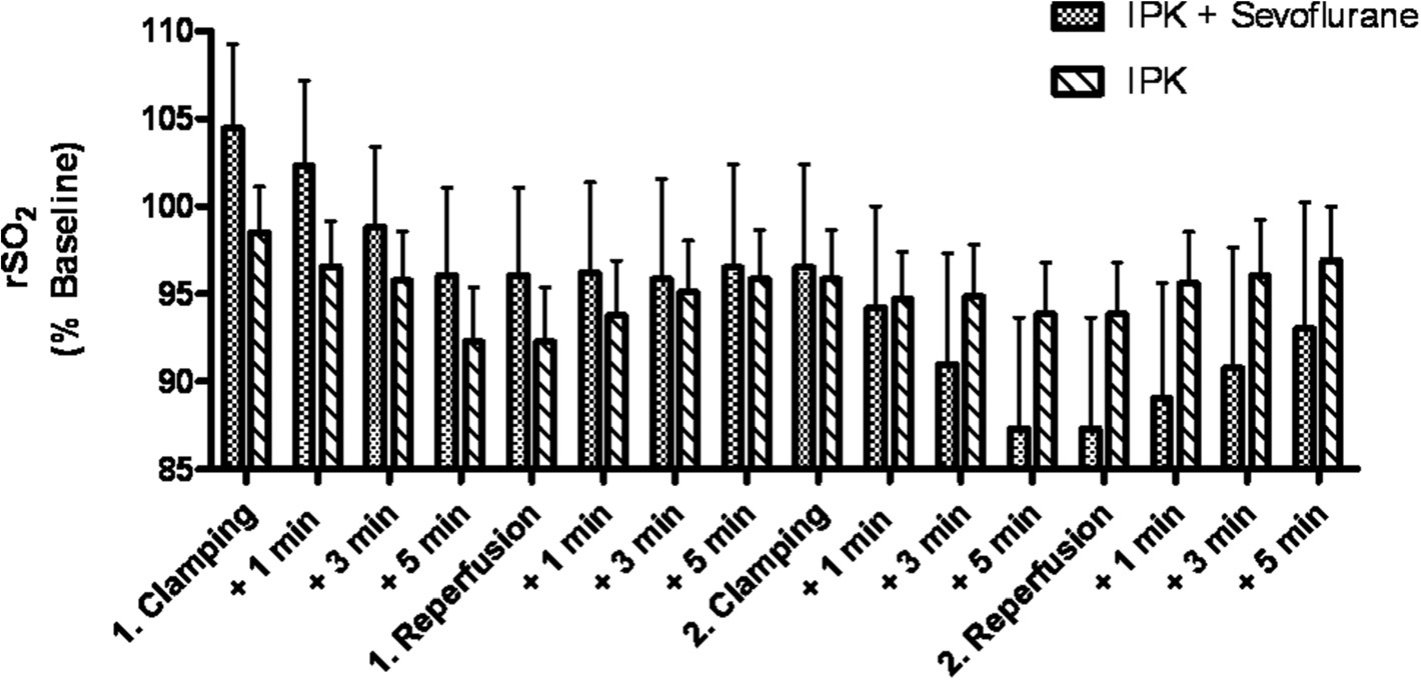
**rSO**_**2 **_**in the anterior tibial muscle during ischemic preconditioning.** rSO_2_ in the anterior tibial muscle during ischemic preconditioning with (IPC + Sevoflurane, n = 20) and without (IPC, n = 20) sevoflurane preconditioning before ischemic preconditioning. There was a significant effect of the factor “time” (p < 0.0001) and the combination of the factors “time” and “sevoflurane” (p = < 0.0001) on rSO_2_. rSO_2_ was normalized to baseline prior to induction of anesthesia to facilitate group comparison.

A significant effect of clamping and declamping during IPC on rSO_2_ was observed and the change in rSO_2_ over time is different for patients with and without sevoflurane. Figure 2 shows rSO_2_ in the anterior tibial muscle during ischemic preconditioning in patients with sevoflurane preconditioning (n = 20) and patients with IPC only (n = 20). There was a significant effect of the factor “time” (time from beginning of cyclic clamping and declamping for IPC, p < 0.0001) and the combination of the factors “time” and “sevoflurane” (patients with sevoflurane preconditioning vs. patients without sevoflurane preconditioning, p = < 0.0001) on rSO_2_ (Table 3). In patients with sevoflurane preconditioning rSO_2_ decreased less during the first clamping and more during the second clamping than in patients with IPC only. There was also a significant difference between the time course of rSO_2_ measured at the leg at which surgery was performed and rSO_2_ measured on the opposite leg (control, no surgery and no IPC) during IPC (p = 0.0007, Table 4). rSO_2_ decreased on the side with IPC, but not on the other side, thus indicating achievement of significant ischemia in comparison to the leg opposite to the side of surgery during IPC by clamping of the femoral artery. Mean rSO_2_ of all patients immediately before ischemic preconditioning was 64% ± 12. Mean rSO_2_ of the sevoflurane group was 66% ± 13 and mean rSO_2_ of the control group was 62% ± 11 (Difference of means not significant). The time course of rSO_2_, however, showed not in all patients a significant decrease and increase (defined as more than twofold the precision of the measurement) as exspected during clamping and reperfusion. A significant decrease during each of both clamping periods and increase during both reperfusion periods by more than twofold the precision of the measurement was observed in 22 patients (11 in the sevoflurane group and 11 in the propofol group).

#### *Clamping of the femoral artery for surgery*

**Figure 3:**
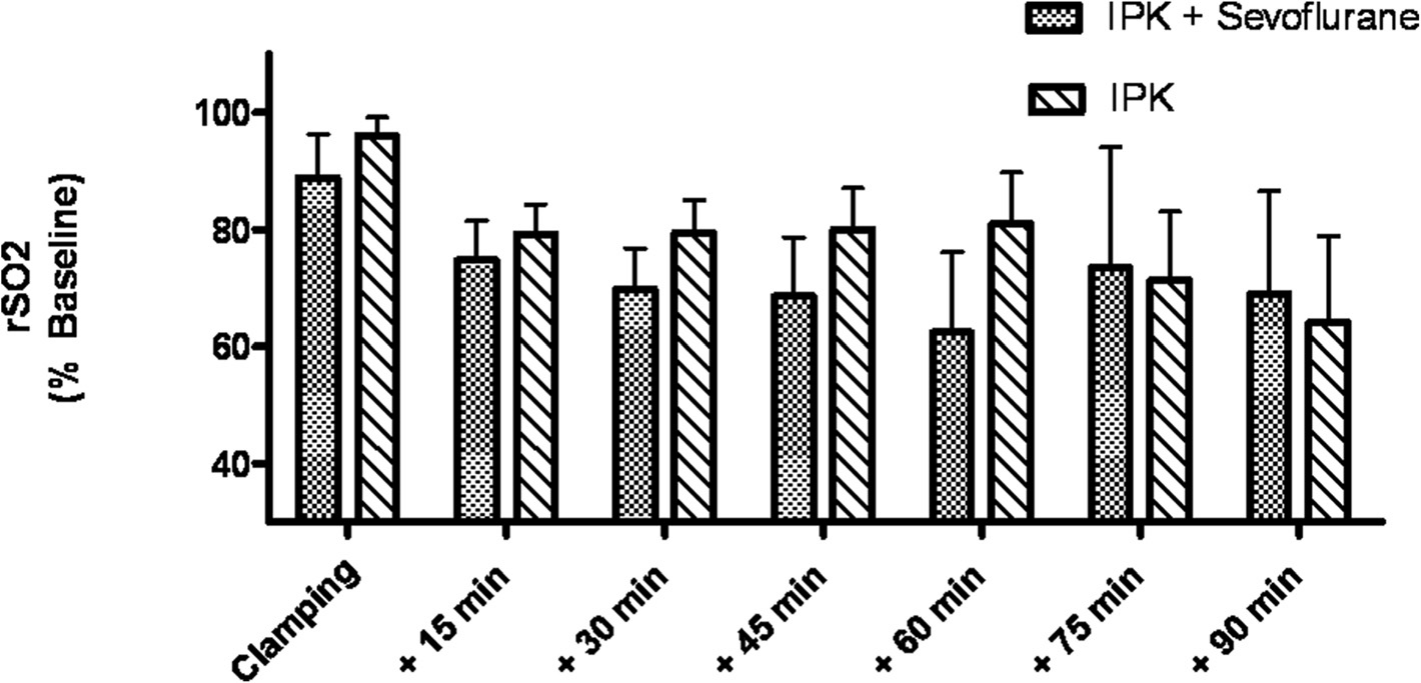
**rSO**_**2 **_**in the anterior tibial muscle during clamping of the femoral artery for surgery.** rSO_2_ in the anterior tibial muscle during clamping of the femoral artery for surgery with (IPC + sevoflurane) and without (IPC) sevoflurane preconditioning before ischemic preconditioning (IPC). There was a significant effect of the factor “time” on rSO_2_ (p < 0.0001), but no significant difference between the groups. rSO_2_ was normalized to baseline prior to induction of anesthesia to facilitate group comparison.

Blood flow due to collateral vessels was present in the clamped artery in all patients. During clamping of the femoral artery for surgery a significant decrease of rSO_2_ was observed in both groups. Figure 3 shows rSO_2_ in the anterior tibial muscle during clamping of the femoral artery for surgery in patients with sevoflurane preconditioning and controls. Immediately before clamping, mean rSO_2_ of the sevoflurane group was 66% ± 13 and mean rSO_2_ of the control group was 61% ± 11 (Difference of means not significant).

There was a significant effect of the factor “time” (p < 0.0001) on rSO_2_, but no significant effect of the factor “sevoflurane” (p = 0.34) and the combination of the factors “time” and “sevoflurane” (p =0.68) on rSO_2_ (Table 3). Time course of rSO_2_ on the side of surgery and on the opposite side (control, without IPC) were significantly different during clamping of the femoral artery (p = 0.0001, Table 4).

#### *Reperfusion after declamping*

**Figure 4:**
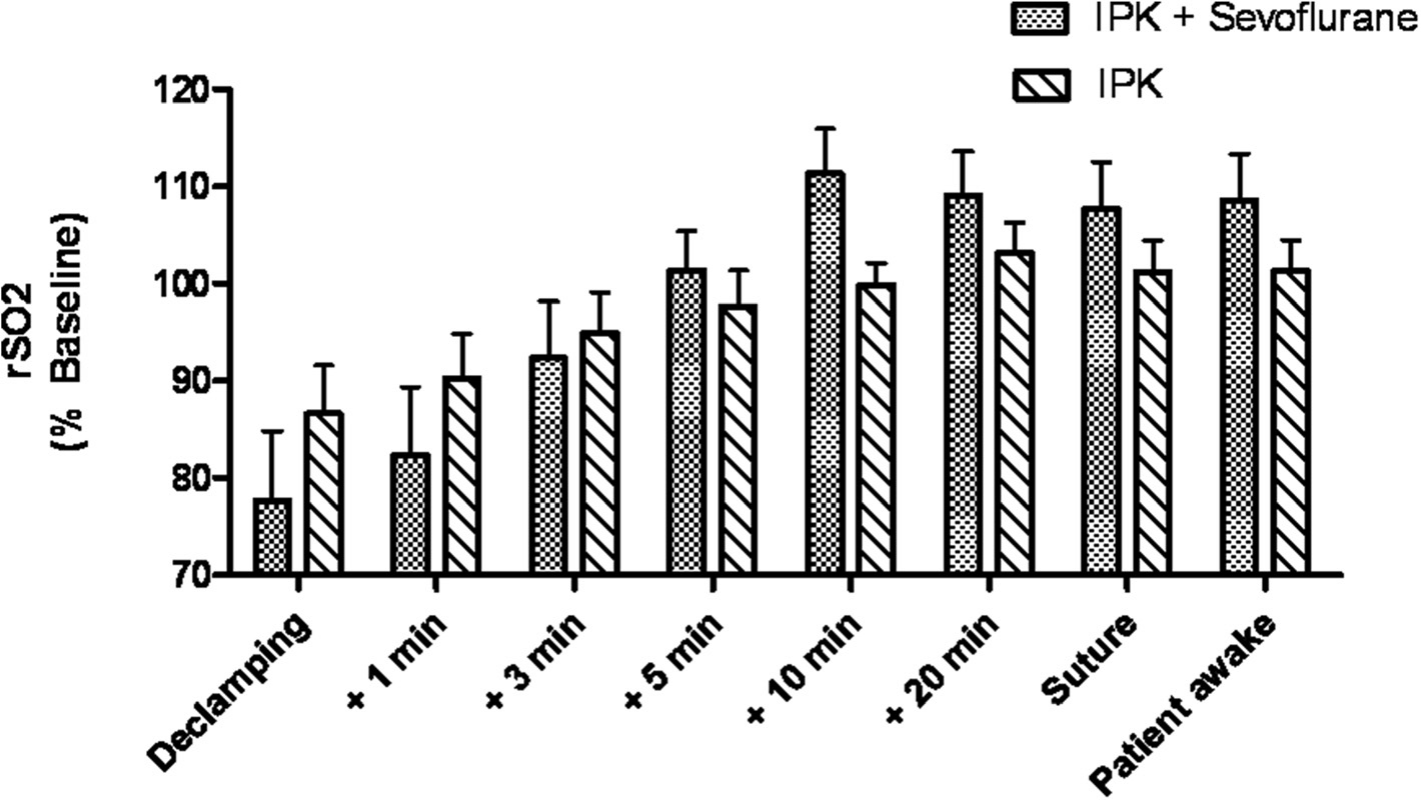
**rSO**_**2 **_**in the anterior tibial muscle after declamping of the femoral artery.** rSO_2_ in the anterior tibial muscle after declamping of the femoral artery in patients with sevoflurane preconditioning (IPC + sevoflurane, n = 20) and without sevoflurane preconditioning (IPC, n = 20) before ischemic preconditioning (IPC). rSO_2_ was normalized to baseline prior to induction of anesthesia to facilitate group comparison.

A significantly faster increase of rSO_2_ was observed in the sevoflurane group in comparison to the group with IPC only during reperfusion after declamping. Figure 4 shows rSO_2_ in the anterior tibial muscle during reperfusion after declamping of the femoral artery in patients with sevoflurane preconditioning and controls. Mean rSO_2_ immediately before declamping was 48% ± 16 in the sevoflurane group and 54% ± 16 in the group with IPC only (Difference of means not significant, p = 0.99).

There was a significant effect of the factor “time” (p < 0.0001) and the combination of the factors “time” and “sevoflurane” (p = 0.008) on rSO_2_ during reperfusion (Table 3). Time course of rSO_2_ on the side of surgery and on the opposite side (control, without IPC) was significantly different after declamping of the femoral artery (p =0.0027, Table 4).

#### *Laboratory values*

Table 5 shows serum hemoglobine, hematocrit, lactate, glucose, potassium, pO_2_, pCO_2_ and base excess in the peripheral venous blood of patients with sevoflurane preconditioning and controls. There was no difference of baseline values before surgery between the groups and no difference of changes in relation to baseline after surgery between the groups.

**Table 5:** Laboratory values

	**IPC + sevoflurane**			**IPC**		
	**(N = 20)**			**(N = 20)**		
**Laboratory value**	**Baseline**	**Post OP**	**p-value**	**Baseline**	**Post OP**	**p-value**
Hemoglobine (mg*dl^-1^)	13.9 ± 2.2	11.5 ± 2.2	< 0.0001	13.8 ± 1.8	11.2 ± 1.4	< 0.0001
Hematokrit (%)	42.6 ± 6.8	34.5 ± 5.7	< 0.0001	42.2 ± 5	34 ± 4	< 0.0001
Lactate (mmol*l^-1^)	1.0 ± 0.3	0.8 ± 0.26	0.07	1 ± 0.3	0.8 ± 0.23	0.008
Glucose (mg*dl^-1^)	118 ± 44.7	104.7 ± 36.2	0.005	113.3 ± 34.6	105.2 ± 25	0.17
Potassium (mmol*l^-1^)	4.2 ± 0.8	4.3 ± 0.7	0.05	3.9 ± 0.4	4.1 ± 0.3	0.02
Base excess (mmol*l^-1^)	0.38 ± 2.01	0.24 ± 2.48	0.71	0.56 ± 1.88	0.02 ± 2.02	0.19

Serum laboratory values of patients undergoing surgical revascularization of occlusive arterial disease with sevoflurane preconditioning before ischemic preconditioning and patients with ischemic preconditioning only (“IPC + sevoflurane” = patients with sevoflurane preconditioning before ischemic preconditioning (IPC), “IPC” = patients without sevoflurane preconditioning before ischemic preconditioning). Differences between patients with sevoflurane preconditioning before IPC and patients with IPC only are not significant (Baseline = preoperative blood sample, Post OP = postoperative blood sample, p-values exploratively without correction for multiple testing).

#### *Heart rate and mean arterial pressure during preconditioning, surgery and reperfusion*

There was no difference between the heart rates of the sevoflurane and control groups. A difference between mean arterial pressure between the sevoflurane group and controls was observed during the first wash-in of sevoflurane preconditioning only. Mean arterial pressure was 79 ± 23 mmHg in the sevoflurane group and 96 ± 23 mmHg in the control group one minute after the beginning of application of sevoflurane (p = 0.01) and the difference remained largely unchanged until the first wash-out. From the beginning of first wash-out no difference of mean arterial pressure was observed between groups.

#### *Clinical outcome data*

We did not observe any difference between the groups regarding postoperative length of hospital stay, major cardiac events, and length of follow up period. Length of hospital stay was 5 (3–9) days in the sevoflurane group and 5 (3–16) days in the group with IPK only (median (range)). Myocardial infarctation occurred in one patient in each group. No fatal outcome was found in the follow up charts. However, there was a wide variety of follow up periods. Longest available follow up period was 1,5 (1–4) years in the sevoflurane group and 1 (0–4) years in the group with IPK only (median (range)). Thus, a fatal outcome shortly after discharge could not be excluded in some cases.

## Discussion

Main results of our prospective randomized interventional pilot study are:

1. Repetitive clamping of the femoral artery for IPC induced a significant repetitive decrease of mean leg muscle rSO_2_ in patients with and without sevoflurane preconditioning.

2. During IPC, a decrease of leg muscle rSO_2_ during each clamping of the femoral artery and a comparable increase of leg muscle rSO_2_ during each reperfusion was observed in 22 of 40 patients.

3. Sevoflurane preconditioning had a significant effect on the time course of leg muscle rSO_2_ during ischemia and reperfusion both during IPC and during postoperative reperfusion.

Peripheral occlusive arterial disease of the lower limb is a frequent disease with an increased risk of death explained by the association with coronary heart disease and cerebral vascular disease. Surgical revascularization by endarterectomy is recommended in approximately 10% of all patients, but amputation can not be avoided in 6.6% of patients and perioperative mortality is 1% [18-20]. Hence, it seemed reasonable to investigate IPC and sevoflurane preconditioning as potential tools to improve outcome after surgical revascularization.

Mean age and gender distribution of the patients in our study were similar to epidemiological studies on the prevalence of peripheral occlusive arterial disease [19]. Mean rSO_2_ baseline values did not differ from values obtained from healthy volunteers probably because most patients had peripheral arterial disease without ischemic symptoms during rest (Fontaine grade II) [21].

### Sevoflurane muscle tissue preconditioning

Sevoflurane protects cardiac tissue by activation of ATP-dependent potassium channels and inhibition of mitochondrial permeability transition pores (MPTP) [22]. ATP-dependent potassium channels are also expressed in peripheral arteries, thus enabling preconditioning with sevoflurane in most tissues [23]. Lucchinetti et al. showed that sevoflurane (0.5 - 1 vol % end-tidal concentration) improved postocclusive hyperemic reaction after 15 minutes of forearm ischemia, thus suggesting a preconditioning effect on limb tissue [23]. Two periods of five minutes of sevoflurane administration (1 MAC) followed by five minutes of washout have been used for effective clinical sevoflurane preconditioning [16,21]. Low subanesthetic sedative concentrations of sevoflurane (<1%) have been shown to be sufficient for pharmacologic preconditioning [24].

In our study, no significant difference of rSO_2_ was observed between groups during sevoflurane preconditioning. However, rSO_2_ values tended to be slightly higher in the sevoflurane group. This is consistent with the known dilatative effect of sevoflurane on the vascular bed.

Sevoflurane preconditioning had a significant effect on the time course of rSO_2_ during IPC. Decrease during the first clamping interval was less prominent in the sevoflurane group than in the control group, but more prominent than in the group with IPC only during the second clamping interval. Albeit not significantly different from the control group, rSO_2_ exceeded baseline values immediately before clamping in the sevoflurane group only. Hence, sevoflurane preconditioning may have dilated collateral vessels of the femoral artery to an extend that rendered a further preconditioning effect by IPC impossible.

There was no difference of rSO_2_ between groups during surgery, but there was a significant effect of sevoflurane on the time course of postoperative reperfusion with higher rSO_2_ in the sevoflurane group at the end of reperfusion.

Taken together, these results suggest successful sevoflurane preconditioning of the skeletal muscle.

### Ischemic preconditioning

Ischemic preconditioning (IPC) has been described in numerous clinical and experimental studies [2]. A study aimed at the efficacy of different preconditioning protocols showed that the efficacy of IPC is probably more dependent on the number of ischemic periodes and reperfusions than on the duration of the periods. Three periods of 2.5, 5 or 10 minutes resulted in improved postischemic muscle function [25]. Clamping during IPC and clamping for surgery led to significant changes of rSO_2_ consistent with cyclic ischemia. In the group without sevoflurane preconditioning, rSO_2_ decreased during the first clamping period and increased during the first reperfusion period. Decrease was less prominent during the second ischemic period, as exspected after successful ischemic preconditioning by the first cycle. Hence, probably IPC had an effect on leg muscle tissue perfusion in our patients. These findings are consistent with previous experimental results showing effective IPC of skeletal muscle. IPC could attenuate capillary flow deficit after ischemia and reperfusion in isolated dog gracilis muscle [26]. Moreover, it has been shown that IPC can reduce infarct size by more than 50% in porcine latissimus dorsi flaps and canine gracilis muscle after ischemia (five hours) and reperfusion (72 and 48 hours respectively) [27,28].

Interestingly, IPC caused significant effects with respect to rSO_2_ in only 22 out of 40 patients. In the remaining patients, collaterals may have prevented complete cessation of blood flow to the lower limb. This points to the fact that an effective IPC may be only achieved in 50% of patients undergoing surgery and thus may render sevoflurane preconditioning an interesting alternative.

Remote preconditioning could also have been applied at sites different from the site of surgery by intermittently inflating a cuff around an extremity. However, preconditioning performed by inflating a cuff around an extremity above systolic blood pressure involves risks like embolism or tissue damage by compression that are probably more predominant in patients with vascular disease. Since in most patients vascular disease is not confined to the extremity at which surgery is performed, the same risks may occur at the other extremities. Hence, we prefered to investigate IPC by clamping the femoral artery with the same clamping technique as applied for definite clamping for surgery [3].

### Heart rate and mean arterial pressure during preconditioning and reperfusion

There was no difference of mean heart rate between the sevoflurane group and controls during the measurement and there was no difference of mean arterial pressure between the sevoflurane group and controls during ischemic preconditioning, surgery and reperfusion. There was a small difference of mean arterial pressure between the sevoflurane group and controls during the first wash-in. This difference was probably caused by the vasodilatative effect of sevoflurane. The difference was compensated by the intravenous application of cafedrin-theodrenalin (Akrinor®) during the following phases of wash-in and wash-out. This lower mean arterial pressure during the first sevoflurane wash-in could have contributed to preconditioning of the leg tissue in principle. However, the difference was small and was observed during the first wash-in only. As described above, optimal preconditioning is achieved by cyclic preconditioning stimuli. Hence, a substantial preconditioning effect due to this difference seems unlikely and the preconditioning effect is regarded as caused by sevoflurane only.

### Limitations

Measurement of rSO_2_ by NIRS is influenced by changes of serum hemoglobine concentration [29]. Serum hemoglobine decreased during surgery by 2.6 g.dl^-1^ without any difference between our study groups. Hence, serum hemoglobine could have biased our results, but to the same extent in both groups, thus having no impact on the differences observed. An influence of pCO_2_ on rSO_2_ during the measurement was excluded by continuously monitoring endtidal pCO_2_ and keeping it constant [30]. Excessive obesity may also distort measurement of muscle tissue oxygenation by thick subcutaneous fat layers, but was not present in our patients [13].

Clinical endpoints were not included in this study, since the patient number was too small to expect significant differences between groups. However, length of hospital stay, major cardiac events and length of follow up period have been obtained from the patients charts. There were no significant differences between the groups regarding these parameters. Due to the large variability of occlusive arterial disease and outcome after surgical revascularization, possibly a substantially higher patient number is needed to measure an effect of preconditioning on clinical endpoints.

## Conclusion

Ischemic preconditioning by clamping of the femoral artery and sevoflurane preconditioning of lower limb muscle tissue during surgical revascularization have significant effects on rSO_2_ in patients with occlusive arterial disease of the lower limb. The results suggest that a large scale prospective randomized trial including an IPC group, a group with sevoflurane preconditioning and a control group on the effect of IPC and sevoflurane preconditioning on outcome after surgical repair of peripheral vascular disease with major morbidity as clinical endpoint should be performed.

## Competing interests

Markus Steinfath, Axel Fudickar, Erol Cavus, Markus Siggelkow, Sarah Kunath and Dana Voß declare that they have no potential conflicts of interest including commercial relationships such as consultation and equity interests.

Prof. B. Bein received honoraria for consulting and giving lectures from Abbvie, the manufacturer of sevoflurane.

## Authors’ contributions

AF and BB designed the study protocol. AF, SK and DV performed the sevoflurane preconditioning, data collection and evaluation. MS performed the ischemic preconditioning. AF, SK, DV, EC, MS and BB were involved in drafting and revising the manuscript. All authors read and approved the final manuscript.

## Pre-publication history

The pre-publication history for this paper can be accessed here:

http://www.biomedcentral.com/1471-2253/14/54/prepub
